# Functional Bladder Paraganglioma Treated by Partial Cystectomy

**DOI:** 10.1155/2019/4549790

**Published:** 2019-11-12

**Authors:** Amine Hermi, Hamza Ichaoui, Aziz Kacem, Houcem Hedhli, Faten Gargouri, Ramzi Khiari, Samir Ghozzi

**Affiliations:** ^1^Faculty of Medicine of Tunis, University Tunis El Manar, Department of Urology, Military Hospital of First Instruction of Tunis, Tunisia; ^2^Faculty of Medicine of Tunis, University Tunis El Manar, Department of Pathology, Military Hospital of First Instruction of Tunis, Tunisia

## Abstract

Paraganglioma is a rare neuroendocrine tumor that arises from the autonomic nervous system. The urinary bladder paraganglioma accounts for less than 0.1% of bladder tumors. It remains a challenging entity to diagnose and treat due to its rareness and the lack of specific symptoms. Treatment modalities include transurethral resection and cystectomy (partial or total). The authors report a new case of an isolated paraganglioma of the urinary bladder in a 52-year-old female patient that underwent partial cystectomy. This case aims to remind the clinical, histological and therapeutic features of this rare tumor.

## 1. Introduction

Pheochromocytoma is a tumor developed from the chromaffin tissues of the sympathetic nervous system. It is commonly localized in the adrenal medulla. However, in about 10% of cases, pheochromocytomas may occur in ectopic or extra adrenal sites, extending from head to pelvis. Ectopic pheochromocytoma is called paraganglioma.

The urinary bladder is a rare localization of paragangliomas, accounting for less than 1% of all pheochromocytomas. It occurs in all age groups, more commonly in females [1].

It can be nonfunctional or functional by catecholamine secreting. It may be difficult for urologists to pinpoint the exact diagnosis.

Histological examination is often the only key leading to diagnosis. This neoplasm is usually benign but can occasionally show malignant behavior.

In presenting this case, we aim to provide a complete overview of etiology, symptom, diagnosis and possible treatments of paraganglioma of the urinary bladder, in order to write up a useful article for urologists that could have to manage similar cases.

## 2. Case Presentation

A 62 year-old-woman, complaining from micturition lipothymia for six months, was admitted in our hospital. She had no significant past medical history, and there was no history of headaches, palpitations, profuse sweating, hypertension or gross hematuria. Her family history was unremarkable.

Physical examination was normal, and blood pressure was within normal range at 120/80 mmHg. Cytobacteriological examination of the urine was negative. The patient was not anemic and the white cell count was within normal range. There was no renal failure.

**Figure 1:**
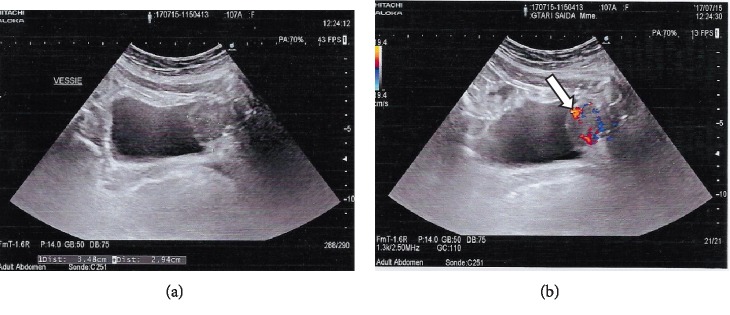
(a) Sagittal gray-scale ultrasound image of the urinary bladder shows a heterogeneous 40 mm mass along the anterior wall of the bladder. (b) Sagittal color Doppler ultrasound image demonstrates marked flow within the bladder mass (arrow).

An abdominal ultrasonographic examination was performed initially, which depicted an approximately 40 mm heterogeneous mass along the anterior wall of the bladder with abundant internal flow at Doppler interrogation (Figure 1).

**Figure 2:**
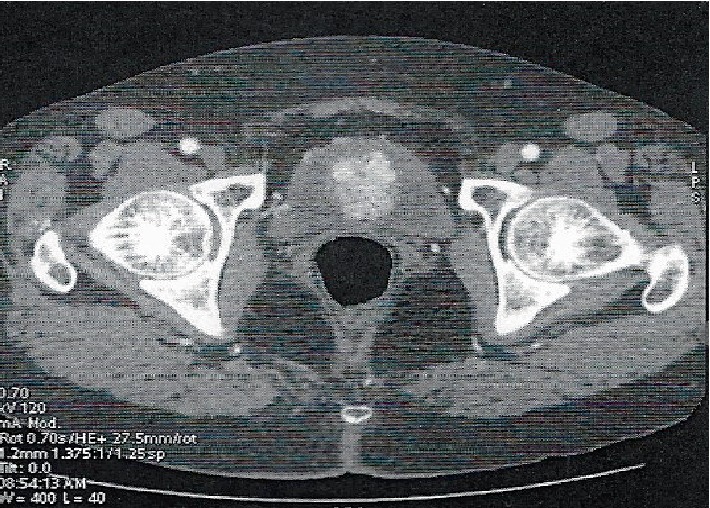
Transverse computer tomography image showing an avidly enhancing mass in the anterior urinary bladder wall.

An abdominal computed tomography (CT) scan, with intravenous administration of contrast medium, revealed a 32 mm heterogenous lobulated anterior bladder wall mass. The mass showed intense uniform enhancement with contrast study. There was no sign of any lymphadenopathy or metastatic invasion (Figure 2).

**Figure 3:**
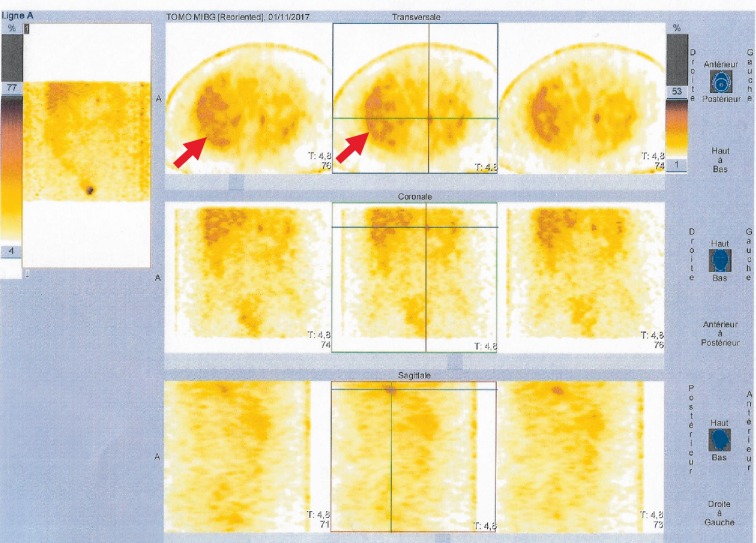
Iodine-123-meta-iodobenzylguanidine (I-123 MIBG) scintiscan showing intense tracer uptake in the anterior side of the bladder consistent with bladder paraganglioma (arrow).

Further imaging with Iodine-123-meta-iodobenzylguanidine (I-123 MIBG) scintiscan confirmed the presence of bladder pheochromocytoma without metastatic disease (Figure 3).

The 24-hour urine metanephrine and normetanephrine measurement was within normal levels.

The patient underwent a complete and deep transurethral resection of the bladder tumor. A solid submucosal mass was seen on the left lateral wall near the neck of the urinary bladder, with normal mucosal covering. The resection was complete and tissue was submitted to histopathological examination. During the procedure, the patient became severely hypertensive. Her blood pressure raised up to 236/118 mmHg and pulse rate dropped to 46/min. This episode was controlled with intraoperative intravenous antihypertensive and atropine. Postoperative recovery was uneventful.

**Figure 4:**
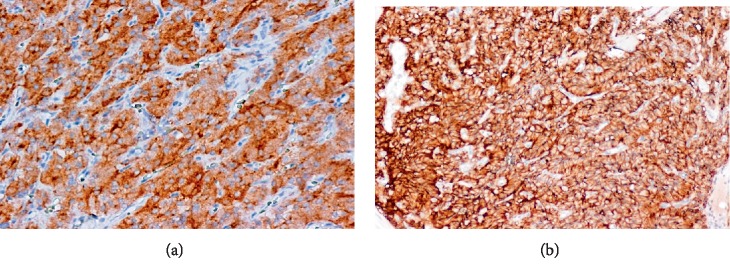
(a) Tumor cells are strongly stained with synaptophysin (×200). (b) Tumorous small cells with a positive immunostaining for anti CD56 (×100).

The histological examination of the tumor confirmed the diagnosis of vesical paraganglioma showing tumorous small cells with a positive immunostaining for synaptophysin and anti-CD 56 (Figure 4).

**Figure 5:**
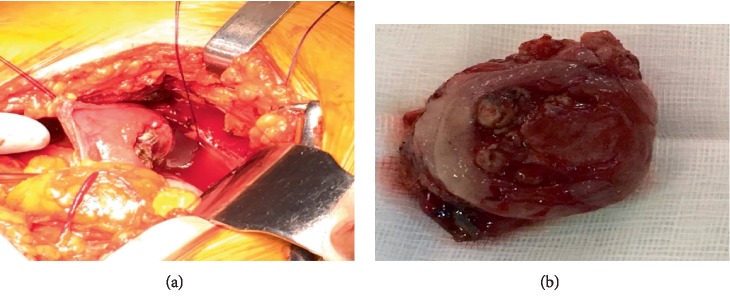
(a) The surgically resected lesion is well defined and polypoid, and it appears to be vasc. (b) Macroscopic view of bladder paraganglioma. The tumour is red-brown in colour, lobulated and well-circumscribed.

The patient underwent partial cystectomy (Figure 5). The surgical margin of the removed specimen was negative. Postoperative recovery was uneventful.

On a recent follow-up at 12 months, her cystoscopy examination did not reveal any evidence of recurrence. Her radiological assessment (thoracic-abdomino-pelvic CT) was negative for metastatic disease.

## 3. Discussion

Paraganglioma or extra-adrenal pheochromocytoma of the urinary bladder arises from chromaffin tissue of the sympathetic nervous system within the layers of the bladder wall. It is a very rare localization of extra renal pheochromocytoma. However, in the genitourinary tract, the urinary bladder is the most common site (79.2%), followed by the urethra (12.7%), pelvis (4.9%), and ureter (3.2%) [2]. Paraganglioma of the urinary bladder was first reported in 1953 by Zimmerman [3], and since that time there have been fewer than 200 cases described.

Bladder pheochromocytoma occurs more frequently in women than in men, and mainly during the third decade of life [4].

It is most commonly situated at the dome or the trigone of the bladder and may be nonfunctional or functional. They remain usually benign, but about 20% of paragangliomas may show malignant behavior [4].

Functional paragangliomas secrete catecholamines, which may lead to symptoms similar to a hyperfunctioning adrenal pheochromocytoma, such as headaches, palpitations, dizziness, and sweating. These symptoms occur by in spells or paroxysm. They are mainly precipitated by micturition, overdistention of the bladder, defecation, sexual activity, ejaculation, or bladder instrumentation. Painless hematuria is the presenting complaint in about 60% of reported cases [4]. However, it has been estimated that 17% of bladder pheochromocytomas patient are hormonally nonfunctional, so less symptomatic.

On most imaging modalities a bladder paraganglioma is indistinguishable from other tumor types specially in ultrasound and CT examination. If the bladder tumor is submucosal, and shows marked enhancement after contrast administration and markedly increased, homogeneous signal intensity on T2-weighted magnetic resonance images. Paraganglioma should be considered in the differential diagnosis, although these tumors usually show less hyperintensity than the typical adrenal pheochromocytoma [5].

Metaiodobenzylguanidine scintigraphy demonstrates high specificity for pheochromocytoma but this nuclear medicine scan is less sensitive compared to magnetic resonance imaging [5].

Surgical resection is the treatment of choice. To avoid hypertensive crisis during the mass manipulation, preoperative treatment with alpha and beta-blocking agents is required, starting 1–3 weeks before surgery [6]. The cystoscopic appearance of a submucosal tumor with an intact surface, yellow at the cut, should raise the suspicion of bladder paraganglioma.

Histological examination of the tumor tissue will confirm diagnosis with diffuse, strong positivity for neuron-specific enolase (NSE), synaptophysin, and/or chromogranin. Histologically, it is often misdiagnosed as urothelial carcinoma, malignant melanoma and granular cell tumor [7].

Between 5% and 15% of the paragangliomas of the urinary bladder are said to be malignant. However, no histological criteria have been established to distinguish between benign and malignant tumors. Only the appearance of local invasion or distant metastases confirms that the tumor is cancerous [8].

Forty gray external beam radiation therapy in patients with paragangliomas has shown clinically significant symptomatic relief for at least one year or until death. For patients in which surgery or local radiation therapy is not viable, palliative system options include 131-iodine-labeled meta-iodobenzylguanidine (131I-MIBG) therapy and cytotoxic chemotherapy [9].

Most of bladder paragangliomas grow slowly and have a good prognosis. Regular follow-up is necessary to evaluate the bladder function after partial cystectomy as well as the surveillance for late recurrences. It must be lifelong and include cystoscopy and imaging study. No consensus has been established for the frequency of these measures: nevertheless, we suggested at least an annual follow-up for those patients who are asymptomatic or whenever clinically indicated [10].

## 4. Conclusions

Paragangliomas are rare tumors of the bladder. They may present clinical, radiology and pathological features similar to bladder cancer. There are typical symptoms due to catecholamine secretion especially associated with micturation periods. They are rarely malignant and most of the tumors are treated successfully with partial cystectomy. Other treatment modalities such transurethral resection can be considered as effective first-line treatments. Moving forward, it would be helpful to standardize the reporting guidelines of pheochromocytomas cases to better understand the natural process and outcomes.
